# Navigating the Labyrinth: Chylothorax and Chylous Ascites Unveiled After Abdominal Surgery for an Exceptionally Rare Tumor

**DOI:** 10.7759/cureus.66239

**Published:** 2024-08-05

**Authors:** Anam Umar, Amber E Faquih, Muhammad Bilal, Jeffery Garner

**Affiliations:** 1 Internal Medicine, Ascension St. Vincent's Birmingham, Birmingham, USA; 2 Infectious Diseases, University of Alabama at Birmingham, Birmingham, USA; 3 Pulmonary and Critical Care, Ascension St. Vincent's Birmingham, Birmingham, USA

**Keywords:** multidisciplinary management, case report, chylothorax, chylous ascites, adrenal schwannoma

## Abstract

Schwannomas, originating from the Schwann sheath of peripheral or cranial nerves, are rare tumors commonly found in the head and neck or extremities. Adrenal schwannomas, however, are exceedingly rare, accounting for less than 1% of all adrenal tumors. Here, we present a case of a 31-year-old Caucasian woman diagnosed with an adrenal schwannoma, which was incidentally discovered during imaging studies for an unrelated issue. Following laparoscopic adrenalectomy, the patient developed chylous ascites (CA) and coexistent chylothorax, posing a diagnostic challenge and necessitating a multidisciplinary approach to management.

## Introduction

Schwannomas are common in the head and neck region or the extremities; visceral involvement of schwannomas is extremely rare. Only a handful of cases of adrenal schwannomas have been reported [1]. Overall, adrenal schwannomas account for less than 1% of all adrenal tumors, 1-3% of all schwannomas, and 0.4% of retroperitoneal tumors [2]. Recent advancements in imaging techniques have led to the inadvertent identification of more adrenal tumors. However, a preoperative diagnosis of adrenal schwannoma is challenging, and determining whether a mass is an adrenal schwannoma can only be definitively established through pathologic examination with immunohistochemical staining [3,4]. Surgical resection, whether open or laparoscopic depending on the mass size, is the primary treatment for adrenal tumors, though it can lead to several complications despite its generally acknowledged safety. Two rare but severe complications that may arise following retroperitoneal surgery are chylothorax and chylous ascites (CA) [5]. We present a rare case of adrenal schwannoma complicated post-operatively by CA and coexistent chylothorax.

## Case presentation

**Figure 1:**
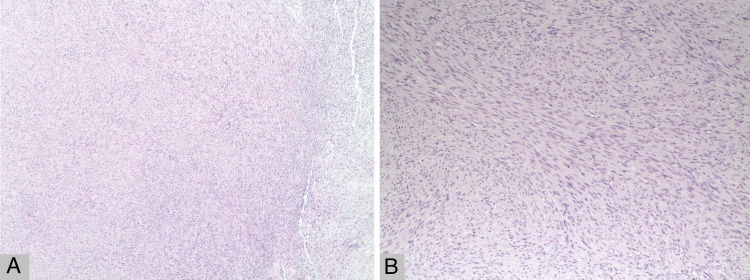
Microscopic examination of adrenal schwannoma A) Histologic examination at low power (4X) reveals a hypercellular tumor composed of spindle cells (hematoxylin & eosin stain). B) High power view (10X) shows elongated and wavy tumor cells with tapered ends and mild nuclear atypia (hematoxylin & eosin stain).

A 31-year-old Caucasian woman with an unremarkable medical history presented to the urology clinic with a left adrenal mass initially discovered several years prior. Despite a previously unremarkable metabolic workup, recent imaging studies revealed an enlargement of the mass with atypical characteristics, prompting further investigation. Routine blood and hormonal tests, including serum cortisol, dehydroepiandrosterone sulfate (DHEAS), metanephrine, and normetanephrine, along with 24-hour urinary metanephrine and normetanephrine, were within normal limits. However, due to the progressive increase in size from 3.8 to 4.4 cm and the atypical features on imaging, a laparoscopic adrenalectomy was deemed necessary and performed. Subsequent histopathological examination identified the mass as a cellular schwannoma (Figure 1), a rare finding in this location. Although the laparoscopic adrenalectomy proceeded smoothly, the patient experienced a transient complication characterized by a milky discharge from one of the surgical incisions, which resolved spontaneously within four days without intervention. A month after discharge from the hospital, she presented with shortness of breath, abdominal distention, and significant weight gain, prompting readmission.

**Figure 2:**
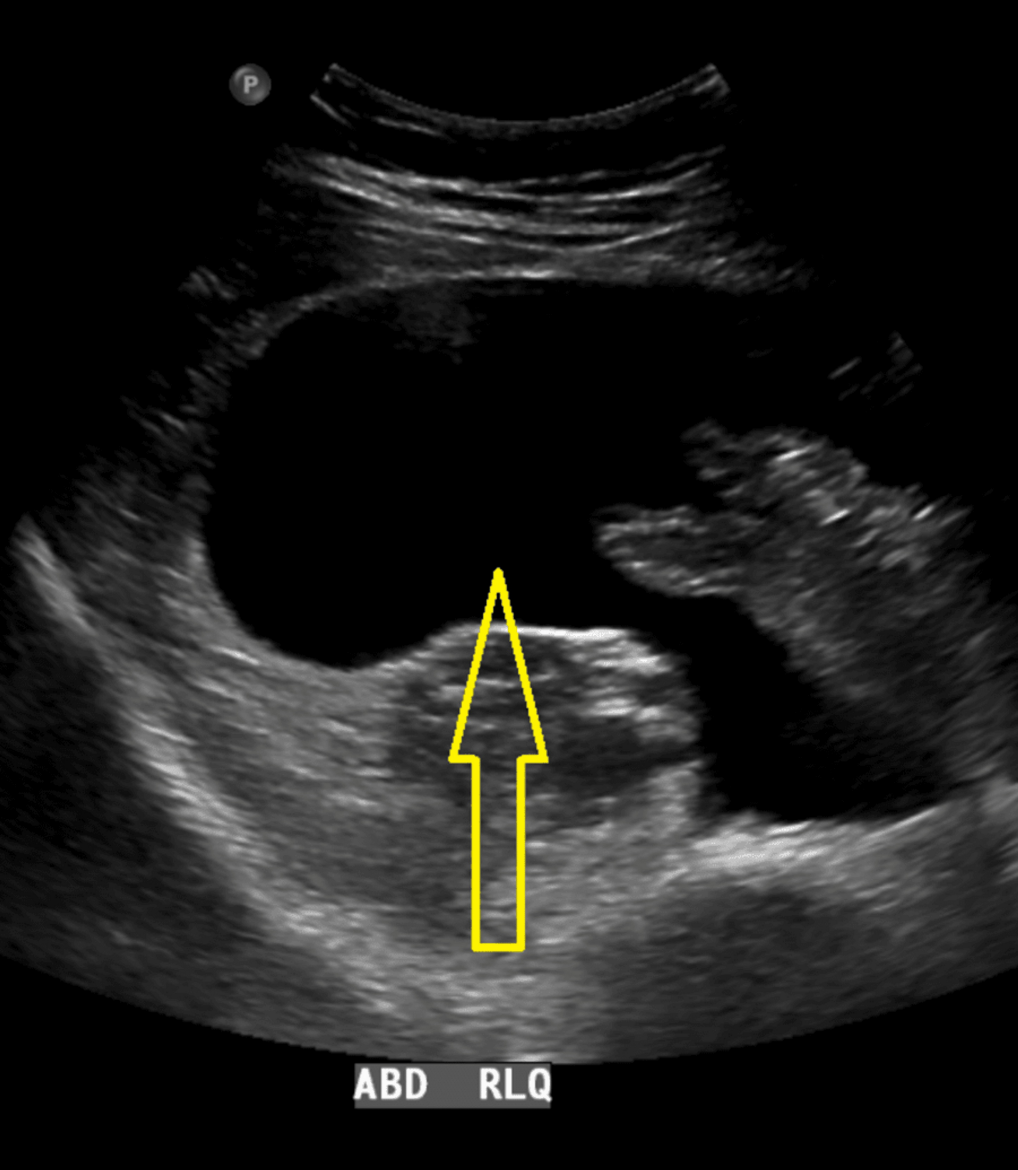
Ultrasound of the abdomen This ultrasound image of the right lower quadrant (RLQ) of the abdomen shows a large anechoic area (indicated by the yellow arrow), suggestive of a fluid-filled structure. The surrounding hyperechoic areas may represent the bowel and adjacent soft tissues.

**Figure 3:**
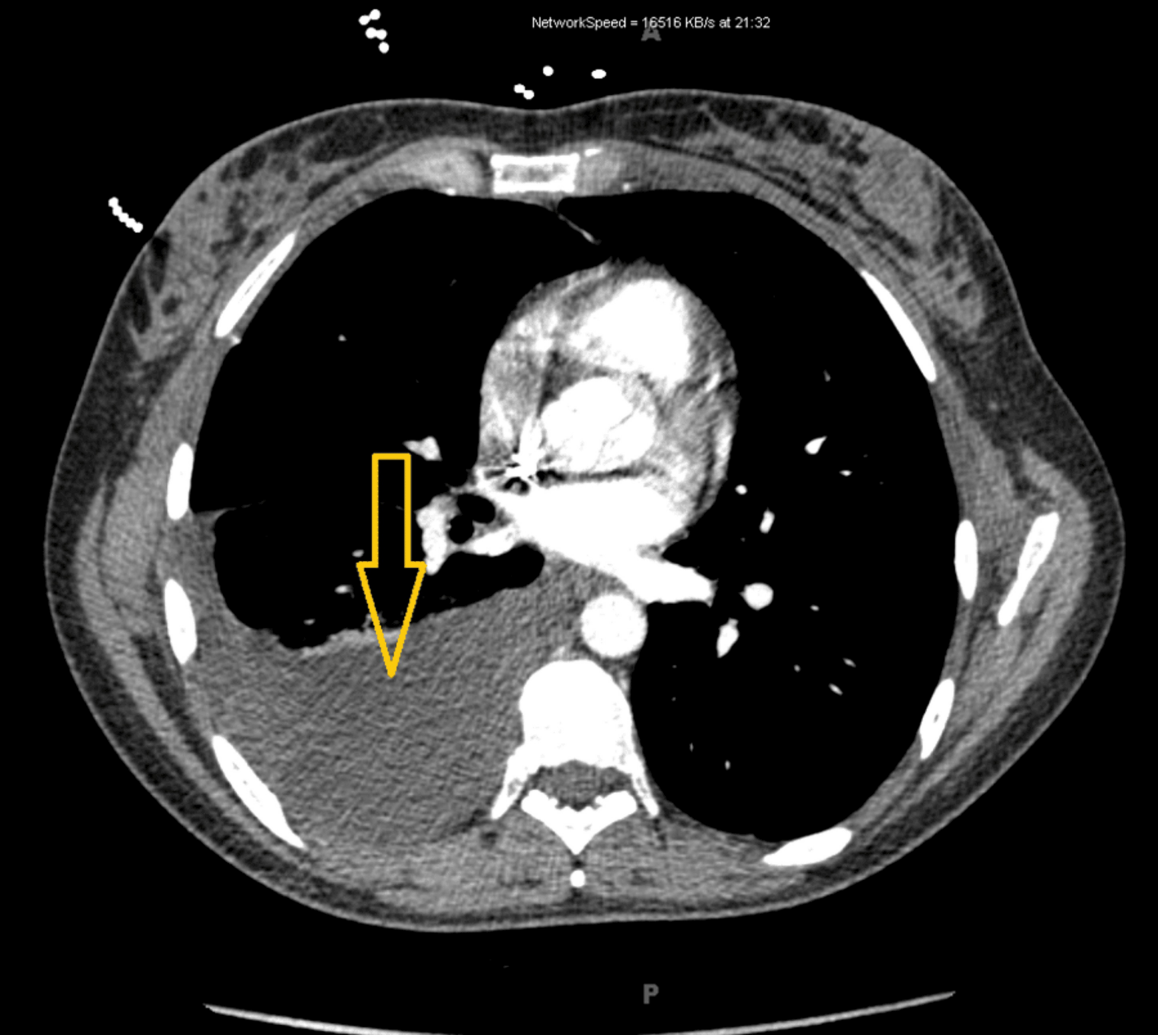
CT scan of the chest This CT scan of the chest shows a large pleural effusion (indicated by the yellow arrow).

**Figure 4:**
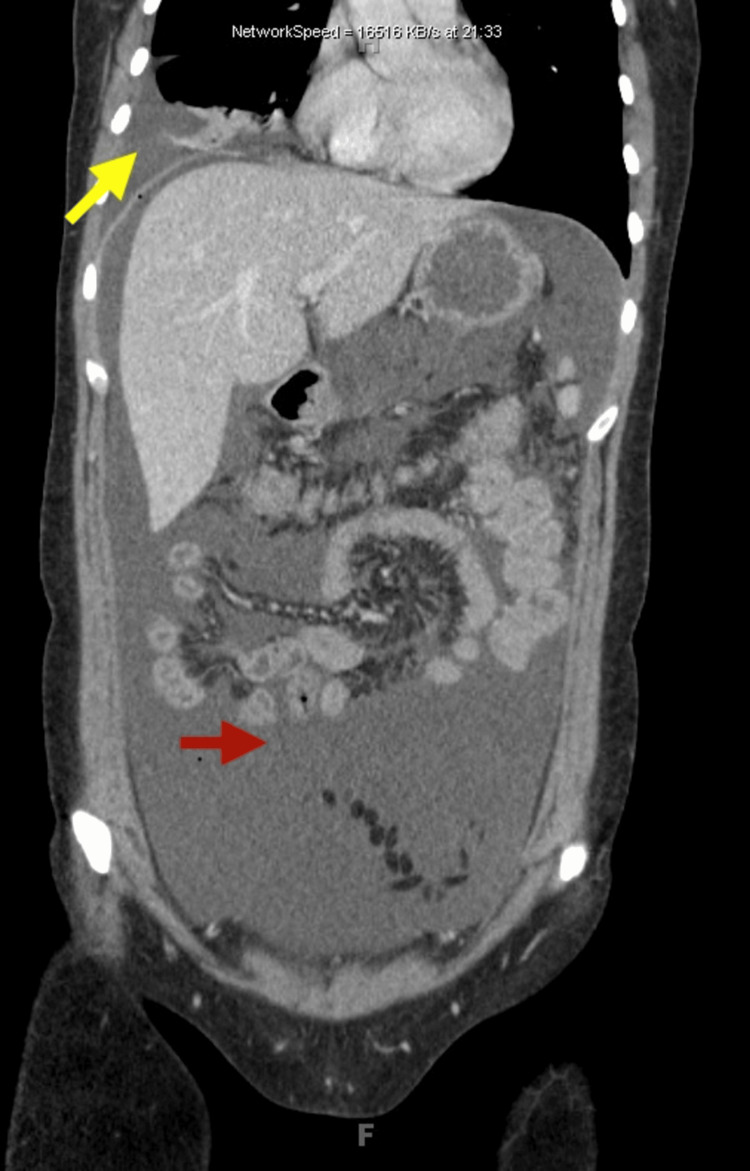
CT scan of the chest, abdomen, and pelvis This CT scan shows a pleural effusion (indicated by the yellow arrow) and ascites (indicated by the red arrow).

**Figure 5:**
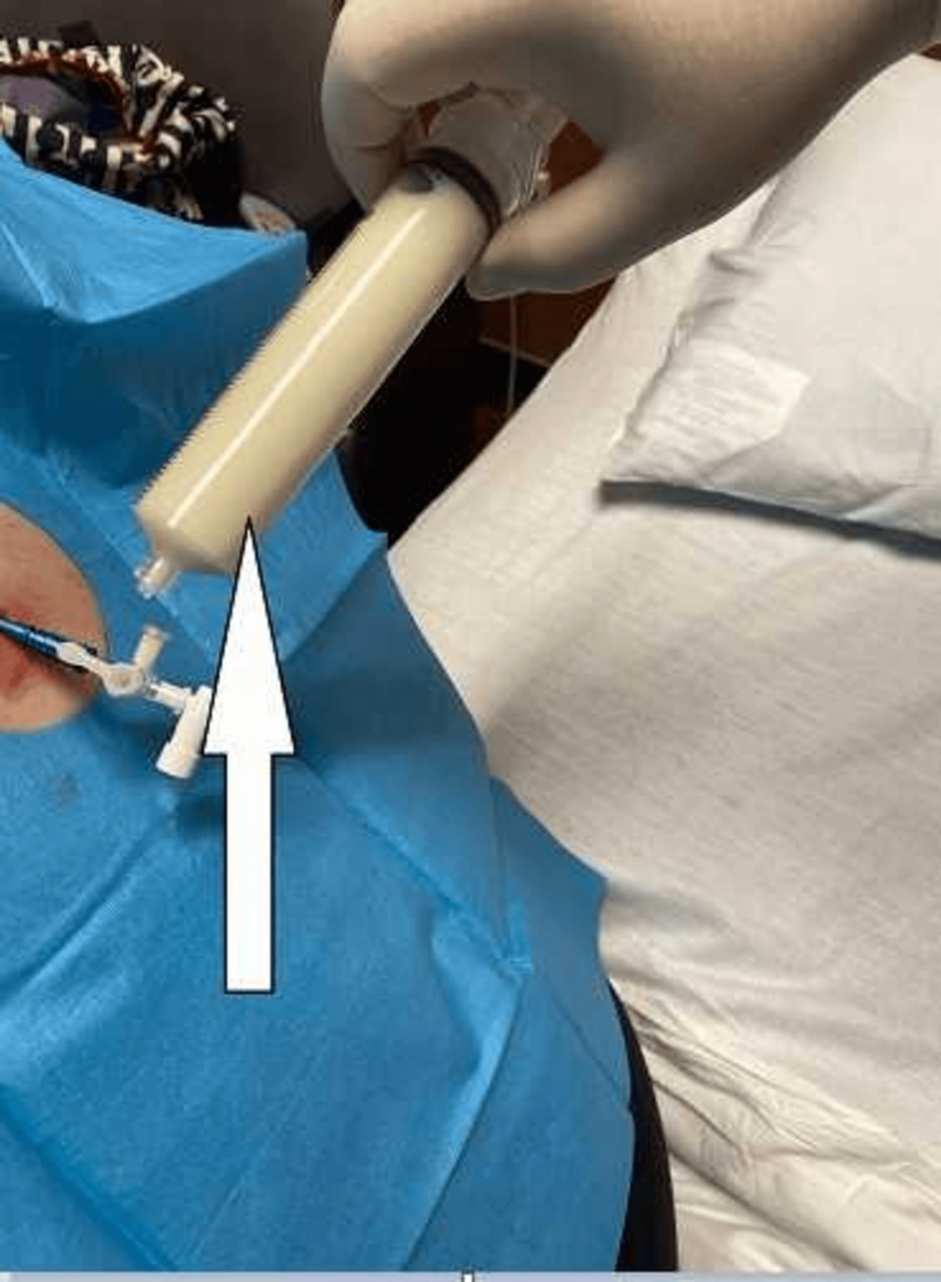
Thoracentesis This image shows the collection of milky white fluid during a thoracentesis procedure. The fluid, which appears chylous, is typically collected from the pleural space to relieve symptoms and for diagnostic evaluation.

On readmission, imaging studies revealed the presence of ascites, omental nodularity, retroperitoneal lymphadenopathy, and a large right-sided pleural effusion, indicative of a potential complication or metastatic spread (Figures 2-4). To address these concerns, therapeutic interventions, including thoracentesis (with the collection of 1.4 L of milky white fluid) and paracentesis (removal of 2.5 L of fluid), were performed, leading to the diagnosis of chylothorax (Table 1, Figure 5) and CA based on body fluid analysis. The patient was started on a high-protein, low-fat diet regimen and initiated on somatostatin analog octreotide therapy to manage her symptoms and potentially mitigate further disease progression. An abdominal drain was placed by interventional radiology to facilitate the removal of accumulated fluid (variable daily output between 30 and 150 cc). Following these interventions, there was a notable improvement in the patient's clinical condition over the next six days, prompting discharge from the hospital. The patient was closely monitored on an outpatient basis for response to treatment and to detect any recurrences.

**Table 1:** Analysis of the milky white fluid obtained after thoracentesis mg/dL (milligrams per deciliter), g/dL (grams per deciliter), cells/mcL (cells per microliter), % (percentage)

Body fluid type	Pleural Fluid
Color	white/milky
Clarity	turbid
Albumin (mg/dL)	2,509
Protein (g/dL)	4.8
Red blood cells (cells/mcL)	3,159
Lymphocytes (%)	66
Triglycerides ((mg/dL)	3,774
Cholesterol (mg/dL)	188
Lactate dehydrogenase (mg/dL)	141
Glucose (mg/dL)	89

## Discussion

Schwannomas are tumors that arise from Schwann cells, which form the myelin sheath surrounding nerve fibers in the peripheral nervous system [6]. Theodore Schwann, a prominent German histologist and physiologist, first characterized Schwann cells (1810-1882). Later, Verocay first described schwannomas in 1908 [7,8], and in 1920, Nils Ragnar Eugène Antoni identified two contrasting patterns within schwannomas [6].

Schwannomas are usually benign [9]. However, it is important to note that 5-18% of schwannomas are linked to von Recklinghausen's disease. When this condition is present, schwannomas tend to be malignant and are more likely to appear in multiple locations.

Adrenal schwannomas are believed to arise from one of the two sets of nerves that innervate the adrenal gland's medulla: the vagus or phrenic nerves. The schwannomas emerge from the medulla and compress the adrenal cortex as they grow [10]. Adrenal schwannomas are more commonly observed in females during the second or fifth decade of life, indicating a tendency toward occurrence in middle-aged women [11]. Typically, these tumors do not exhibit any symptoms and are found unexpectedly during imaging studies conducted for unrelated purposes.

Retroperitoneal schwannomas are often incidentally discovered during evaluations for nonspecific abdominal pain, typically exceeding 4 cm in size at diagnosis. Imaging of a retroperitoneal mass localized to the adrenal region commonly presents a range of potential differentials, including adrenocortical adenoma, pheochromocytoma, myelolipoma, adrenocortical carcinoma, ganglioneuroma, and adrenal metastasis. Surgical excision remains the gold standard treatment for retroperitoneal schwannomas depending on the size, providing both diagnostic confirmation and symptom relief [12-14].

As discussed above, CA and chylothorax are rare but serious complications that can occur after retroperitoneal surgery [5]. Postoperative CA arises due to inadvertent intraoperative injury to the lymphatic system, resulting in the leakage of lymph into the peritoneal cavity. The diaphragm then plays a crucial role in facilitating the absorption of CA into the thorax, leading to chylothorax [15,16].

Regarding treatment, in cases like ours, it is advisable to pursue a conservative approach involving a diet high in protein and low in fat, particularly rich in medium-chain triglycerides (MCTs). MCTs are absorbed directly by intestinal cells and transported as free fatty acids (FFA) and glycerol to the liver via the portal vein, bypassing the thoracic duct [17]. Continuous intravenous high-dose somatostatin or its subcutaneous analog octreotide effectively reduces chyle formation in postoperative lymphatic leaks [5]. Alternatively, orlistat, an inhibitor of lipase activity, also shows promise in managing CA [18]. Other treatment options include peritoneal and pleural taps, chest tube drainage, and abdominal drain placement, as performed in our case [17]. Surgical repair is typically considered after a four-week trial of conservative management, with peritoneovenous shunting reserved for refractory cases [19]. When chyle reaccumulates rapidly in the pleural space despite conservative measures and abdominal surgical repair is not feasible, obliteration of the pleural space should be considered [20].

## Conclusions

Adrenal schwannomas are rare, and when surgical intervention is necessary, they can lead to several complications. In our patient's case, chyle leakage resulted in chylothorax and CA, stemming from damage to the lymphatic ducts during retroperitoneal exploration. It is crucial to promptly recognize these complications, which are characterized by milky discharge from surgical sites, abdominal distention, and pleural effusion leading to shortness of breath. Managing these issues effectively requires a multidisciplinary approach, including dietary adjustments, medication with somatostatin analogs, and procedures such as thoracentesis and paracentesis. This all-encompassing care strategy is essential for alleviating symptoms, fostering recovery, and improving patient outcomes.
